# Association of prenatal antibiotics with measures of infant adiposity and the gut microbiome

**DOI:** 10.1186/s12941-019-0318-9

**Published:** 2019-06-21

**Authors:** Mingyu Zhang, Moira K. Differding, Sara E. Benjamin-Neelon, Truls Østbye, Cathrine Hoyo, Noel T. Mueller

**Affiliations:** 1 Department of Epidemiology, Johns Hopkins Bloomberg School of Public Health, Baltimore, MD USA; 2 Department of Health, Behavior and Society, Johns Hopkins Bloomberg School of Public Health, Baltimore, MD USA; 3 Department of Community and Family Medicine, Duke University Medical Center, Durham, NC USA; 4 Department of Biological Sciences and Center for Human Health and the Environment, North Carolina State University, Raleigh, NC USA; 5 Welch Center for Prevention, Epidemiology and Clinical Research, Johns Hopkins University, Baltimore, MD USA

**Keywords:** Gut microbiome, Pediatric obesity, Antibiotic, Child health, Prenatal exposure, Pregnancy

## Abstract

**Background:**

Prenatal antibiotic exposure has been associated with an altered infant gut microbiome composition and higher risk of childhood obesity, but no studies have examined if prenatal antibiotics simultaneously alter the gut microbiome and adiposity in infants.

**Method:**

In this prospective study (Nurture: recruitment 2013–2015 in North Carolina, United States), we examined in 454 infants the association of prenatal antibiotic exposure (by any prenatal antibiotic exposure; by trimester of pregnancy; by number of courses; by type of antibiotics) with infant age- and sex-specific weight-for-length z score (WFL-z) and skinfold thicknesses (subscapular, triceps, abdominal) at 12 months of age. In a subsample, we also examined whether prenatal antibiotic exposure was associated with alterations in the infant gut microbiome at ages 3 and 12 months.

**Results:**

Compared to infants not exposed to prenatal antibiotics, infants who were exposed to any prenatal antibiotics had 0.21 (95% confidence interval [CI] 0.02, 0.41) higher WFL-z at 12 months, and 0.28 (95% CI 0.02, 0.55) higher WFL-z if they were exposed to antibiotics in the second trimester, after adjustment for potential confounders, birth weight, and gestational age. We also observed a dose-dependent association (P-value for trend = 0.006) with infants exposed to ≥ 3 courses having 0.41 (95% CI 0.13, 0.68) higher WFL-z at 12 months. After further adjustment for delivery method, only second-trimester antibiotic exposure remained associated with higher infant WFL-z (0.27, 95% CI 0.003, 0.54) and subscapular skinfold thickness (0.49 mm, 95% CI 0.11, 0.88) at 12 months. Infants exposed to second-trimester antibiotics versus not had differential abundance of 13 bacterial amplicon sequence variants (ASVs) at age 3 months and 17 ASVs at 12 months (false discovery rate adjusted P-value < 0.05).

**Conclusions:**

Prenatal antibiotic exposure in the second trimester was associated with an altered infant gut microbiome composition at 3 and 12 months and with higher infant WFL-z and subscapular skinfold thickness at 12 months.

**Electronic supplementary material:**

The online version of this article (10.1186/s12941-019-0318-9) contains supplementary material, which is available to authorized users.

## Background

Despite efforts to prevent obesity, the prevalence of childhood obesity has more than tripled in the past 40 years [1]. It reached a historical high of 18.5% in 2015–2016 in the United States (US) [2]. Given that one in seven children ages 2–5 years are obese [2], identifying ways to prevent obesity from earlier life stages is needed. One compelling target for the prevention of early life obesity is the infant gut microbiome [3]. Prenatal antibiotic exposure has been shown to disturb the colonization and maturation of the infant microbiome [3, 4]. Recently, there has been growing interest in understanding whether prenatal antibiotics alter child growth and adiposity. While findings have been inconsistent regarding the association of any antibiotic exposure in pregnancy with child obesity, many showed a positive association of second-trimester antibiotic exposure with child weight [5–8]. However, no studies have examined whether prenatal antibiotics simultaneously affect adiposity and the gut microbiome in infancy. To address this gap, we examined associations of prenatal antibiotics with infant weight and adiposity measures at 12 months and, for mechanistic insight, explored associations with the infant gut microbiome at ages 3 and 12 months.

## Methods

Nurture is a prospective birth cohort that recruited mothers from 2013 to 2015 in Durham, North Carolina, US. A detailed description of the cohort and data collection process is provided elsewhere [9, 10]. We included singleton infants born after 28 weeks gestation with no congenital abnormalities. We obtained written informed consent from each mother at recruitment and again shortly after delivery. In this analytic dataset, we included 454 of the 666 mother-infant dyads that had at least one measurement of infant weight-for-length *z* score (WFL-z) or skinfold thickness at 12 months.

We determined maternal and infant oral antibiotic intake from questionnaires and confirmed for those with medical records data. We categorized antibiotics based on the Anatomical Therapeutic Chemical classification system [11]: tetracyclines (J01A); beta-lactam antibacterials, penicillins (J01C); other beta-lactam antibacterials (J01D); sulfonamides and trimethoprim (J01E); macrolides, lincosamides, streptogramins (J01F); quinolone antibacterial (J01M); aminoglycoside antibacterials (J01G); and other antibacterials (J01X). Infant weight, length, and skinfold (subscapular, triceps, abdominal) thicknesses were measured by trained home visitors at 12 months. We calculated the age- and sex-specific WFL-z using the World Health Organization Child Growth Standards [12]. The sum of subscapular and triceps skinfold thicknesses (SS + TR) represents overall adiposity and the ratio of subscapular to triceps skinfold thicknesses (SS/TR) represents central adiposity.

Confounders were defined as variables both related to the exposure and the outcome but not in the causal pathway. Pre-selected potential confounders included maternal age at delivery (continuous variable), race (Black or African American; White; others), marital status (married or living with a partner; others), education (≤ high school graduate; some college; ≥ college graduate), annual household income (≤ $20,000; $20,001 to $40,000; ≥ $40,001), smoked during pregnancy (yes; no), and maternal pre-pregnancy body mass index (BMI, calculated as weight (kg)/[height (m)]^2^, continuous variable). Other covariates included delivery method (vaginal delivery; cesarean delivery), infant sex (male; female), birth weight (continuous variable), and gestational age (continuous variable). All covariates were extracted from or calculated based on questionnaires at recruitment and during home visits.

The primary outcome was infant WFL-z. Secondary outcomes included subscapular, triceps, and abdominal skinfold thicknesses, SS + TR, and SS/TR. We used multivariable linear regression models to estimate the difference in these outcomes at 12 months by any prenatal antibiotic exposure (yes; no), timing of exposure (trimester of pregnancy), number of courses, and types of antibiotics, adjusting for potential confounders (Model 1). We then added infant birth weight and gestational age (Model 2), and further added infant delivery method into the regression models (Model 3). We conducted sensitivity analyses by further adjusting for infant antibiotic intake in the first year of life in Model 1, and by examining if maternal antibiotic use in the first year after delivery associated with infant WFL-z at 12 months. We considered a two-sided P-value < 0.05 as statistically significant.

We also collected fresh stool at 3 months (in 68 infants) and 12 months (in 50 infants) and conducted 16S rRNA sequencing of the V4 region (see Additional file 1: Materials for full microbiome methods). We then examined the association of prenatal antibiotic exposure with differential microbial relative abundance, using beta-binomial regression models and Wald tests, accounting for sequencing depth and within-sample taxa correlation [13]. In these analyses, we used the false discovery rate (FDR) adjustment for multiple comparisons and two-sided FDR-adjusted P-value < 0.05 was defined as statistically significant. We conducted all analyses using Stata 15.1 (StataCorp, College Station, TX) and R 3.5.1 (R Foundation for Statistical Computing, Vienna, Austria).

## Results

Of the 454 mothers included in the analysis, 217 (47.80%) took ≥ 1 course of antibiotics during pregnancy; 77 (16.96%) took ≥ 3 courses; and 60 (13.22%), 69 (15.20%), and 146 (32.16%) took antibiotics during the first, second, and third trimester, respectively. Mothers who took antibiotics during pregnancy had higher pre-pregnancy BMI and were more likely to take antibiotics in the first year after delivery. Their infants were also more likely to have received antibiotics in the first year of life (Table 1).

**Table 1:** Baseline maternal/household and infant characteristics by prenatal antibiotic exposure (n = 454)

Variable, n (%)^a^	Prenatal antibiotic exposure		P-value
	No	Yes	
Number of participants	237	217	
Maternal/household characteristics			
Age at delivery, years, mean (SD)	27.58 (6.15)	28.27 (5.53)	0.22
Pre-pregnancy BMI, kg/m^2^, mean (SD)	28.41 (7.63)	32.47 (10.58)	< 0.001
Race			0.58
Black or African American	157 (66.5%)	148 (68.2%)	
White	50 (21.2%)	49 (22.6%)	
Others	29 (12.3%)	20 (9.2%)	
Married or living with a partner			0.70
Yes	139 (58.6%)	132 (60.8%)	
No	98 (41.4%)	85 (39.2%)	
Education			0.30
≤ High school graduate	102 (43.0%)	103 (47.5%)	
Some college	93 (39.2%)	70 (32.3%)	
≥ College graduate	42 (17.7%)	44 (20.3%)	
Annual household income			0.95
≤ $20,000	138 (59.7%)	129 (60.8%)	
$20,001 to $40,000	44 (19.0%)	41 (19.3%)	
≥ $40,001	49 (21.2%)	42 (19.8%)	
Smoked during pregnancy			1.00
Yes	36 (15.6%)	33 (15.6%)	
No	195 (84.4%)	178 (84.4%)	
Type of breastfeeding			0.13
Exclusive breastfeeding	18 (7.8%)	10 (4.8%)	
Exclusive formula	46 (20.0%)	56 (26.9%)	
Mixed feeding method	166 (72.2%)	142 (68.3%)	
Maternal antibiotic use in the first year after delivery			0.001
Yes	67 (28.3%)	93 (42.9%)	
No	170 (71.7%)	124 (57.1%)	
Infant characteristics			
Sex			0.64
Female	117 (49.4%)	112 (51.6%)	
Male	120 (50.6%)	105 (48.4%)	
Gestational age, week, mean (SD)	38.74 (1.38)	38.46 (1.72)	0.05
Birth weight, kilogram, mean (SD)	3.20 (0.50)	3.23 (0.53)	0.59
Infant antibiotic use in the first year of life			0.01
Yes	84 (35.4%)	102 (47.0%)	
No	153 (64.6%)	115 (53.0%)	

*SD* standard deviation, *BMI* body mass index

^a^Unless otherwise indicated

Table 2 shows the association between prenatal antibiotic exposure and infant WFL-z at 12 months. After adjusting for potential confounders (Model 1), infants exposed to prenatal antibiotics had 0.23 (95% CI 0.02, 0.43) higher WFL-z at 12 months, compared to infants who were not exposed. There was also a dose-dependent association by courses of prenatal antibiotics (P-value for trend = 0.006): compared to infants not exposed, infants who were exposed to 1, 2, or ≥ 3 courses of prenatal antibiotics had 0.09 (95% CI − 0.19, 0.37), 0.19 (95% CI − 0.10, 0.48), or 0.40 (95% CI 0.11, 0.68) higher WFL-z at 12 months, respectively. While there were no significant associations of any prenatal antibiotic exposure with infant skinfold thicknesses, SS + TR, or SS/TR at 12 months after adjusting for confounders (Additional file 2: Table S1), we found that ≥ 3 courses of prenatal antibiotics was associated with 0.68 (95% CI 0.12, 1.24) mm increase in infant abdominal skinfold thickness at 12 months.

**Table 2:** Association (with 95% confidence interval) of prenatal antibiotics and infant weight-for-length *z* score at 12 months

	N	Model 1: adjusted for potential confounders^a^	Model 2: model 1 + infant birth weight + infant gestational age	Model 3: model 2 + delivery method	Sensitivity analysis: model 1 + infant antibiotic intake in the first year
N		410	408	405	410
Any prenatal antibiotic exposure					
No	237	Reference group			
Yes	217	0.23 (0.02, 0.43)	0.21 (0.02, 0.41)	0.14 (− 0.09, 0.37)	0.22 (0.02, 0.43)
Number of courses during the prenatal period					
0	237	Reference group			
1	73	0.09 (− 0.19, 0.37)	0.10 (− 0.17, 0.38)	0.07 (− 0.21, 0.35)	0.09 (− 0.20, 0.37)
2	67	0.19 (− 0.10, 0.48)	0.13 (− 0.15, 0.42)	0.05 (− 0.28, 0.37)	0.19 (− 0.10, 0.48)
≥ 3	77	0.40 (0.11, 0.68)	0.41 (0.13, 0.68)	0.33 (0.02, 0.63)	0.39 (0.11, 0.68)
Timing of prenatal antibiotic exposure					
Reference group: infants not exposed to antibiotics in each specific time period					
First trimester	60	0.21 (− 0.09, 0.51)	0.16 (− 0.13, 0.45)	0.15 (− 0.14, 0.44)	0.21 (− 0.09, 0.50)
Second trimester	69	0.28 (0.002, 0.55)	0.28 (0.02, 0.55)	0.27 (0.003, 0.54)	0.27 (− 0.001, 0.55)
Third trimester	146	0.17 (− 0.05, 0.38)	0.15 (− 0.06, 0.37)	0.04 (− 0.21, 0.29)	0.16 (− 0.05, 0.38)
First year after delivery (maternal intake)	160	0.05 (− 0.17, 0.26)	− 0.02 (− 0.23, 0.18)	− 0.05 (− 0.26, 0.16)	0.05 (− 0.17, 0.26)
Type^b^ of antibiotic exposure					
Reference group: infants not exposed to each specific type of antibiotic					
Beta-lactam antibacterials, penicillins	30	0.10 (− 0.30, 0.49)	0.08 (− 0.31, 0.46)	0.08 (− 0.31, 0.46)	0.10 (− 0.30, 0.49)
Other beta-lactam antibacterials	146	0.19 (− 0.02, 0.41)	0.16 (− 0.05, 0.37)	0.03 (− 0.24, 0.29)	0.19 (− 0.02, 0.41)
Sulfonamides and trimethoprim	15	− 0.05 (− 0.58, 0.48)	− 0.02 (− 0.53, 0.49)	− 0.04 (− 0.55, 0.47)	− 0.06 (− 0.59, 0.47)
Macrolides, lincosamides, streptogramins	46	0.23 (− 0.11, 0.56)	0.23 (− 0.10, 0.56)	0.21 (− 0.12, 0.53)	0.23 (− 0.11, 0.56)
Aminoglycoside antibacterials	23	0.34 (− 0.10, 0.78)	0.32 (− 0.11, 0.75)	0.24 (− 0.19, 0.68)	0.33 (− 0.11, 0.78)
Other antibacterials	69	0.35 (0.08, 0.63)	0.34 (0.08, 0.61)	0.32 (0.05, 0.59)	0.35 (0.08, 0.63)

^a^Potential confounders included maternal age at delivery, race, marital status, educational, annual household income, smoke during pregnancy, and pre-pregnancy body mass index

^b^Tetracyclines (J01A) and quinolone antibacterial (J01M) were excluded due to the small number of people who took them (n < 10)

Associations remained when we further adjusted for infant birth weight and gestational age at birth (Model 2) or infant antibiotic intake in the first year of life (sensitivity analysis). When we further added delivery method to Model 2, only second-trimester antibiotic intake remained associated with infant WFL-z at 12 months (0.27, 95% CI 0.003, 0.54) (Model 3). The second trimester was also the only period in which antibiotic exposure was associated with higher 12-month subscapular skinfold thickness [0.50 (95% CI 0.12, 0.89) mm increase].

**Fig. 1:**
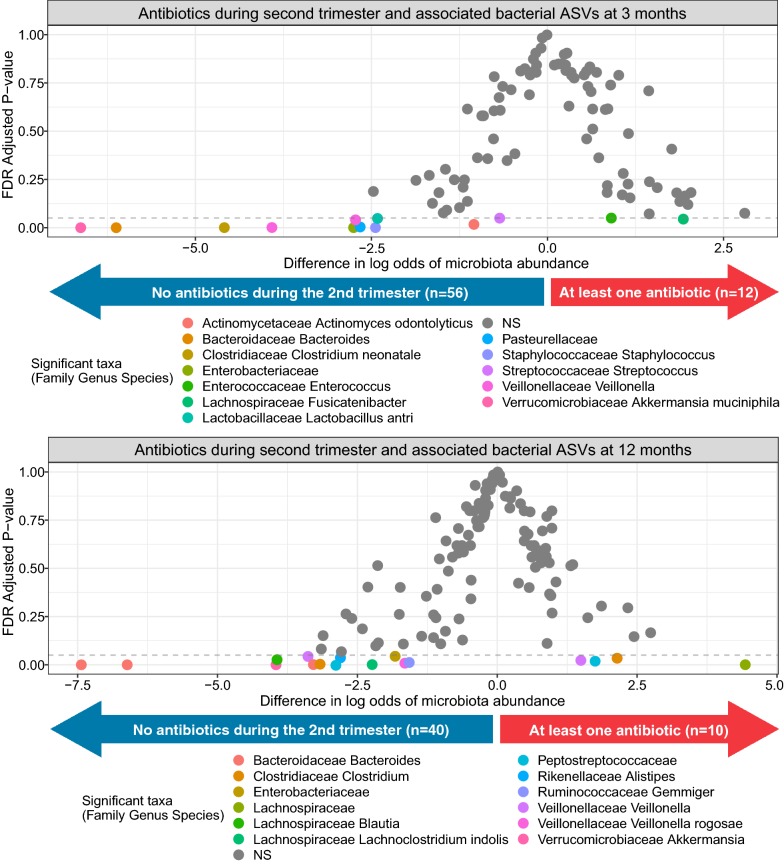
Association of second-trimester antibiotic exposure and infant gut microbiome at 3 and 12 months. The graph shows the estimated differences in log odds of expected bacterial ASV (microbiota) relative abundance in the stool of 3-month-old infants either exposed (n = 12) versus not exposed (n = 56) to antibiotics during the second trimester (top panel) and 12-month-old infants either exposed (n = 10) versus not exposed (n = 40) to antibiotics during the second trimester (bottom panel). The highlighted circles below the dashed line indicate bacterial ASVs significantly associated (FDR-adjusted P-value < 0.05) with antibiotic exposure in the second trimester. Detailed information of these ASVs can be found in Additional file 3: Table S2. ASV indicates amplicon sequence variant; FDR, false discovery rate

Given the persistent association of second-trimester antibiotic exposure with infant 12-month WFL-z, we next examined in a subsample of infants whether second-trimester antibiotic exposure was associated with the infant gut microbiome. Infants exposed to antibiotics in the second trimester versus not exposed had significantly different relative abundance of 13 bacterial amplicon sequence variants (ASVs) at age 3 months and 17 ASVs at 12 months (Fig. 1). Detailed information of these ASVs can be found in Additional file 3: Table S2.

## Discussion

In our low-income, multi-racial prospective birth cohort from North Carolina, we found a dose-dependent association between number of prenatal antibiotic courses and higher infant WFL-z at 12 months, after controlling for potential confounders. After further controlling for delivery mode, only second-trimester antibiotic exposure remained associated with infant weight and adiposity measures at 12 months. Analysis of the infant gut microbiome showed that prenatal antibiotic exposure in the second trimester was associated with differential abundance of 13 unique bacterial ASVs at age 3 months and 17 ASVs at 12 months.

Our group previously reported an association between antibiotic exposure in pregnancy and higher risk of child obesity at 7 years [5]. Several studies have found similar associations in children ages 2 years [7] and 7–16 years [6], while others did not find an association in children ages 3 years [14], 5 years [15], or 4 or 7 years [8]. Consistent with our current study, many have found stronger associations for repeated exposure [6–8] and for exposure in the second trimester [5–8]. In our current study, we showed that second-trimester antibiotic exposure was associated not only with infant weight but also with greater subscapular skinfold thickness at 12 months of age.

The second trimester of pregnancy, when the fetus and fetal gut are rapidly developing [16], may be a particularly susceptible window for immunometabolic programming by the maternal microbiome. We showed that antibiotic exposure in the second trimester is associated with differential abundance of 13 and 17 ASVs in the infant gut microbiome at 3 and 12 months, respectively. The abundance of some taxa remained different over time, including higher abundance of bacteria in the *Lachnospiraceae* family, which has been associated with greater child weight [17], and lower abundance of *Akkermansia muciniphila*, a bacterium associated with metabolic health [18]. An experimental murine model [19] and an observational study in infants delivered preterm [20] also found that prenatal antibiotic exposure is associated with an altered infant microbiome composition. Other studies have found trimester-specific associations of antibiotics with fetal adipokines and birth weight [21], and observed that antibiotic exposure in the second trimester is associated with higher risk of childhood obesity [5–8]. It is possible that antibiotic-induced changes to the maternal microbiota and their metabolites [22] may underlie these trimester-specific associations, but future studies, with stool and blood samples from both mothers and infants, are still needed to test this hypothesis.

There are several strengths of our study, including the confirmation of antibiotic records from the medical records, which reduced recall bias. These records also provided detailed information on the time prescribed, number of courses, and antibiotic type. Moreover, unlike previous studies, we used maternal antibiotic intake in the first year after delivery for comparison to see if the association is specific to pregnancy. In these analyses, maternal antibiotic intake in the first year after delivery was not associated with infant WFL-z at 12 months, implying that the association of prenatal antibiotics with infant weight is not due to maternal propensity for infection.

Some limitations also exist. Although we controlled for important confounders, we cannot rule out the possibility of residual confounding or confounding by indication for the antibiotic prescription (e.g., underlying infection). Moreover, we were not able to confirm maternal compliance with these prescriptions, thus misclassification of the prenatal antibiotic exposures might exist. However, such misclassification would most likely to be non-differential by outcome and, if any, bias the association towards the null. Furthermore, we only collected stool from a subsample of infants, and we did not collect stool or vaginal samples from the mother to test whether antibiotic-induced changes to the microbiome mediated the observed associations with measures of adiposity.

## Conclusions

Our findings suggest that prenatal antibiotic exposure in the second trimester is associated with an altered infant gut microbiome composition at 3 and 12 months and higher infant WFL-z and subscapular skinfold thickness at 12 months. Larger birth cohorts with repeated microbiome measures are needed to confirm our findings.

## Additional file

**Additional file 1.** Materials for full microbiome methods.

**Additional file 2: Table S1.** Adjusted association (with 95% confidence interval) of prenatal antibiotics and infant skinfold thicknesses at 12 months.

**Additional file 3: Table S2.** Associations of second trimester antibiotics and bacterial ASV (microbiota) abundance as shown in Fig. 1.

## Data Availability

The datasets used in this study are potentially available from Dr. Benjamin-Neelon with appropriate ethical and legal agreements in place.

## Abbreviations

US: United States
WFL-z: weight-for-length z score
FDR: false discovery rate
BMI: body mass index
SS: subscapular skinfold thickness
TR: triceps skinfold thickness
ASV: amplicon sequence variant
