# Supplementary material for: Association of prenatal antibiotics with measures of infant adiposity and the gut microbiome
Source: Ann Clin Microbiol Antimicrob. 2019 Jun 21;18:18. doi: 10.1186/s12941-019-0318-9 (PMC6587281; doi:10.1186/s12941-019-0318-9)
Supplement: Supplementary file 1 — Additional file 1. Materials for full microbiome methods. [file 12941_2019_318_MOESM1_ESM.pdf]

Additional File 1: Materials for Full Microbiome Methods

Design of primers

Primers used for 16S sequencing are identical to those of a previously published procedure by Kozich and colleagues[1].

DNA Extraction

Stool specimens were extracted using the QIAgen MagAttract PowerSoil for KingFisher (Qiagen, Venlo, Netherlands). 0.5 g feces were deposited in each well of the bead plate of the Powersoil kit and extracted according to the manufacturer's instructions. DNA extracts were quantified using the Quant-iT dsDNA high sensitivity kit (ThemoFisher, Waltham, MA).

16S rRNA gene PCR amplicon sequencing

Polymerase chain reaction (PCR) amplification was done using 1-10 ng of extracted DNA with the Thermo Phusion Hot Start II DNA Polymerase (ThermoFisher Cat. No. F549S). PCR cycle conditions were 98°C for 2 minutes, followed by 30 cycles of 98°C for 20 seconds, 55°C for 15 seconds, and 72°C for 30 seconds, and 72°C for 10 minutes following the cycling steps. Library preparation was done using a previously published standard operating procedures[1] with the details and product information outlined below. 10 µl of the final product was used to normalize with the SepalPrep Normalization Prep Plate Kit (ThermoFisher Cat. No. A1051001) to 1-2 ng/µl and 5 µl of each normalized sample was pooled into a single library per 96-well plate. Library pools were further concentrated using the DNA Clean and Concentrator kit (Zymo Cat. No. D4013). A dilution series was performed for each of the pooled libraries for subsequent quality control steps. Each pool was analyzed using the Agilent Bioanalyzer using the High Sensitivity DS DNA assay (Agilent Cat. No. 5047-4626) to determine approximate fragment size, and to verify library integrity. Library pools with unintended amplicons were purified using the Qiagen QIAquick Gel Extraction Kit (Qiagen Cat. No. 28706). Pooled library concentrations were determined using the KAPA Library Quantification Kit for Illumina (KAPA Cat. No KK4824). Library pools were diluted to 4 nM and denatured into single strands using fresh 2.0 N NaOH. The final libraries were loaded at 8 pM, with an additional PhiX spike-in of 20%. The amplicon library was sequenced on the MiSeq using the MiSeq 500 Cycle V2 Reagent Kit (Illumina Cat. No. MS-102-2003).

16S rRNA gene amplicon sequence preprocessing

The V4 region of the 16S rRNA gene was sequenced using an Illumina MiSeq and demultiplexed according to sample-specific barcodes as described in Kozich et al[1]. We completed read quality

control and the resolution of amplicon sequence variants (ASV) using the DADA2 R package (v1.8.0)[2] with the following choices and parameters. Per DADA2 authors' recommendations[3], we trimmed all sequences at fixed positions following inspection of aggregate positional sequence quality in order to omit severe drops in quality due to end effects (Figure S1). We trimmed forward reads before position 10 and after position 240 (5' and 3' ends, respectively) and reverse reads before position 10 and after position 150 (5' and 3' ends, respectively). We applied filters to remove individual reads from the dataset if they contained any ambiguous bases, mapped to any part of the phiX genome, or were predicted based on quality values to have a maximum expected error greater than 2 ('filterAndTrim' function[2]).

16S rRNA gene amplicon sequence denoising

We calculated error model estimation on a randomly-selected subset of 106,431,120 bases in 462,744 reads from 12 samples out of 118 total from the run using the 'learnErrors' function[2]. We applied this aggregate model to every sample during final pooled amplicon sequence denoising via the 'dada' function[2], with other parameters set to their default values. After denoising, we merged forward and reverse reads with at least 12 bp overlap and no mismatches using the 'mergePairs' function[2]. We performed chimera detection and removal via the 'pooled' method in the 'removeBimeraDenovo' function[2] (783 non-chimeric sequence variants out of 7250, ~81.7% of reads non-chimeric). We classified the taxonomy of each amplicon sequence independently via the DADA2 'assignTaxonomy' function[2] using the HITdb v.1.00 16S rRNA gene sequence database for human intestinal samples[4] formatted for and available on the DADA2 website[5]. We aligned ASVs using the R DECIPHER package[6] and a maximum likelihood phylogenetic tree was computed using a generalized time-reversible with Gamma rate variation model with optimization of the proportion of invariable sites and gamma rate parameter, via the phangorn package[7], as described in a recently published workflow[8]. We combined the resulting tree with the table of ASV counts for each sample and merged with experimental design data for loading into the phyloseq package[9].

Differential Abundance

We conducted differential abundance testing of bacterial ASVs between conditions with the Wald test on expected relative abundances using beta-binomial regression for correlated count data, as implemented in the R package corncob[10,11]. A multiple testing correction (Benjamini-Hochberg/FDR) was applied to the statistical significance of each result. Two-sided FDR-adjusted P-value <0.05 was defined as significant.

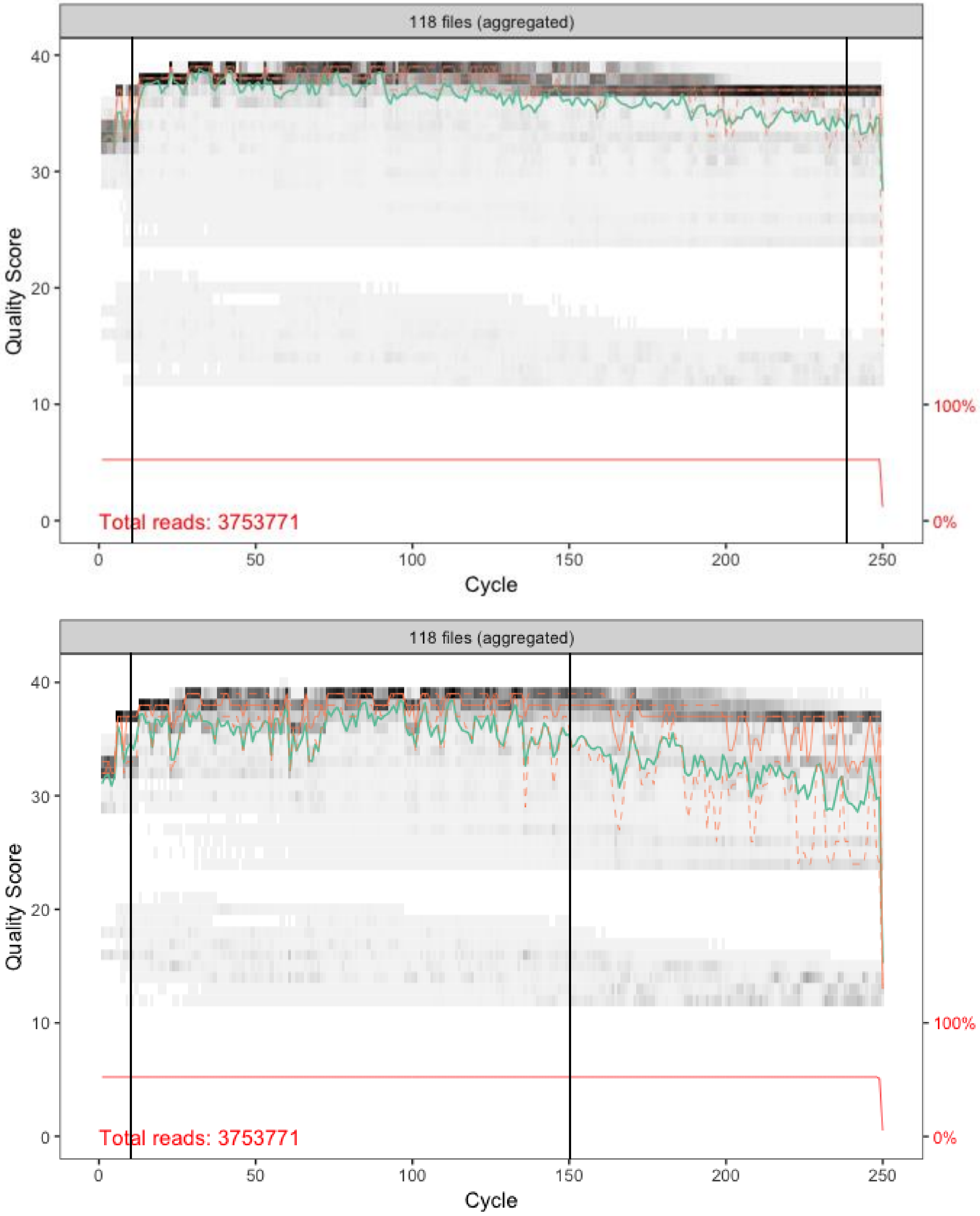

Figure S1. Aggregate positional quality of 16S rRNA amplicon sequence reads. Forward and reverse reads are separated into top and bottom panels, respectively. The left and right trimming positions for the reads are indicated with a vertical black line

References for Supplementary Material

1. Kozich JJ, Westcott SL, Baxter NT, Highlander SK, Schloss PD. Development of a dual-index sequencing strategy and curation pipeline for analyzing amplicon sequence data on the MiSeq Illumina sequencing platform. *Applied and Environmental Microbiology.* 2013;79(17):5112-5120.
2. Callahan BJ, McMurdie PJ, Rosen MJ, Han AW, Johnson AJ, Holmes SP. DADA2: High-resolution sample inference from Illumina amplicon data. *Nature Methods.* 2016;13(7):581-583.
3. Callahan B. DADA2 Pipeline Tutorial (1.8). https://benjjneb.github.io/dada2/tutorial.html. Accessed September 28, 2018.
4. Ritari J, Salojarvi J, Lahti L, de Vos WM. Improved taxonomic assignment of human intestinal 16S rRNA sequences by a dedicated reference database. *BMC Genomics.* 2015;16:1056.
5. Callahan B. Taxonomic reference data. https://benjjneb.github.io/dada2/training.html. Accessed September 25, 2018.
6. Wright ES. Using DECIPHER v2.0 to Analyze Big Biological Sequence Data in R. *The R Journal.* 2016;8(1):352-359.
7. Schliep KP. phangorn: phylogenetic analysis in R. *Bioinformatics.* 2011;27(4):592-593.
8. Callahan BJ, Sankaran K, Fukuyama JA, McMurdie PJ, Holmes SP. Bioconductor Workflow for Microbiome Data Analysis: from raw reads to community analyses. *F1000Research.* 2016;5:1492.
9. McMurdie PJ, Holmes S. phyloseq: an R package for reproducible interactive analysis and graphics of microbiome census data. *PloS One.* 2013;8(4):e61217.
10. Martin BD, Willis A, Witten D. Count Regression for Correlated Observations with the Beta-binomial. https://github.com/bryandmartin/corncob. Accessed September 27, 2018.
11. Martin BD, Witten D, Willis AD. Modeling microbial abundances and dysbiosis with beta-binomial regression. *arXiv e-prints.* 2019. https://ui.adsabs.harvard.edu/abs/2019arXiv190202776M. Accessed April 24, 2019.
