# Supplementary material for: Association of prenatal antibiotics with measures of infant adiposity and the gut microbiome
Source: Ann Clin Microbiol Antimicrob. 2019 Jun 21;18:18. doi: 10.1186/s12941-019-0318-9 (PMC6587281; doi:10.1186/s12941-019-0318-9)
Supplement: Supplementary file 2 — Additional file 2: Table S1. Adjusted association (with 95% confidence interval) of prenatal antibiotics and infant skinfold thicknesses at 12 months. [file 12941_2019_318_MOESM2_ESM.pdf]

Additional File 2

Table S1 Adjusted* association (with 95% confidence interval) of prenatal antibiotics and infant skinfold thicknesses at 12 months

	Subscapular skinfold thickness (SS)	Triceps skinfold thickness (TR)	Abdominal skinfold thickness	Overall adiposity (SS+TR)	Central adiposity (SS/TR)
**N**	412	411	410	411	411
**Any prenatal antibiotic exposure**					
No			Reference group		
Yes	0.10 (-0.19, 0.39)	-0.02 (-0.41, 0.38)	0.24 (-0.16, 0.65)	0.10 (-0.51, 0.71)	0.01 (-0.02, 0.04)
**Number of courses during the prenatal period**					
0			Reference group		
1	-0.08 (-0.48, 0.32)	-0.29 (-0.84, 0.26)	0.15 (-0.41, 0.71)	-0.36 (-1.20, 0.49)	0.02 (-0.03, 0.06)
2	0.02 (-0.40, 0.43)	0.07 (-0.50, 0.64)	-0.11 (-0.68, 0.47)	0.10 (-0.77, 0.98)	-0.01 (-0.06, 0.04)
≥3	0.36 (-0.04, 0.76)	0.18 (-0.37, 0.73)	0.68 (0.12, 1.24)	0.56 (-0.29, 1.40)	0.02 (-0.02, 0.07)
**Timing of prenatal antibiotic exposure**					
Reference group: Infants not exposed to antibiotics in each specific time period					
First trimester	0.27 (-0.15, 0.69)	0.15 (-0.42, 0.73)	0.49 (-0.10, 1.08)	0.43 (-0.46, 1.32)	0.01 (-0.03, 0.06)
Second trimester	0.50 (0.12, 0.89)	0.16 (-0.38, 0.69)	0.48 (-0.07, 1.03)	0.67 (-0.15, 1.49)	0.04 (-0.00, 0.09)
Third trimester	0.09 (-0.22, 0.39)	-0.002 (-0.42, 0.42)	-0.03 (-0.46, 0.40)	0.10 (-0.55, 0.75)	0.001 (-0.03, 0.04)
First year after delivery (maternal intake)	-0.01 (-0.30, 0.29)	-0.08 (-0.50, 0.33)	0.10 (-0.32, 0.52)	-0.07 (-0.71, 0.56)	0.005 (-0.03, 0.04)

* Adjusted for potential confounders including maternal age at delivery, race, marital status, educational, annual household income, smoke during pregnancy, and pre-pregnancy body mass index.
