# Supplementary material for: Association of prenatal antibiotics with measures of infant adiposity and the gut microbiome
Source: Ann Clin Microbiol Antimicrob. 2019 Jun 21;18:18. doi: 10.1186/s12941-019-0318-9 (PMC6587281; doi:10.1186/s12941-019-0318-9)
Supplement: Supplementary file 3 — Additional file 3: Table S2. Associations of second trimester antibiotics and bacterial ASV (microbiota) abundance as shown in Fig. 1. [file 12941_2019_318_MOESM3_ESM.pdf]

Additional File 3

Table S2 Associations of second trimester antibiotics and bacterial ASV (microbiota) abundance as shown in Figure 1

Significant microbiota at 3 months

Beta*	SE	FDR-adjusted P-value	Phylum	Class	Order	Family	Genus	Species
-6.63	0.98	< 0.001	Verrucomicrobia	Verrucomicrobiae	Verrucomicrobiales	Verrucomicrobiaceae	Akkermansia	muciniphila
-2.75	0.68	< 0.001	Proteobacteria	Gammaproteobacteria	Enterobacteriales	Enterobacteriaceae	NA	NA
-0.68	0.30	0.049	Firmicutes	Bacilli	Lactobacillales	Streptococcaceae	Streptococcus	NA
0.91	0.40	0.050	Firmicutes	Bacilli	Lactobacillales	Enterococcaceae	Enterococcus	NA
-6.13	0.82	< 0.001	Bacteroidetes	Bacteroidia	Bacteroidales	Bacteroidaceae	Bacteroides	NA
1.93	0.84	0.044	Firmicutes	Clostridia	Clostridiales	Lachnospiraceae	Fusicatenibacter	NA
-2.66	0.81	0.003	Proteobacteria	Gammaproteobacteria	Pasteurellales	Pasteurellaceae	NA	NA
-2.73	1.16	0.040	Firmicutes	Negativicutes	Selenomonadales	Veillonellaceae	Veillonella	NA
-1.04	0.39	0.017	Actinobacteria	Actinobacteria	Actinomycetales	Actinomycetaceae	Actinomyces	odontolyticus
-4.59	1.10	< 0.001	Firmicutes	Clostridia	Clostridiales	Clostridiaceae	Clostridium	neonatale
-2.44	0.55	< 0.001	Firmicutes	Bacilli	Bacillales	Staphylococcaceae	Staphylococcus	NA
-3.91	1.05	0.001	Firmicutes	Negativicutes	Selenomonadales	Veillonellaceae	Veillonella	NA
-2.41	1.06	0.048	Firmicutes	Bacilli	Lactobacillales	Lactobacillaceae	Lactobacillus	antri

Significant microbiota at 12 months

Beta*	SE	FDR-adjusted P-value	Phylum	Class	Order	Family	Genus	Species
-1.83	0.79	0.044	Proteobacteria	Gammaproteobacteria	Enterobacteriales	Enterobacteriaceae	NA	NA
-3.96	0.91	< 0.001	Verrucomicrobia	Verrucomicrobiae	Verrucomicrobiales	Verrucomicrobiaceae	Akkermansia	NA
1.49	0.58	0.023	Firmicutes	Negativicutes	Selenomonadales	Veillonellaceae	Veillonella	NA
-7.43	1.16	< 0.001	Bacteroidetes	Bacteroidia	Bacteroidales	Bacteroidaceae	Bacteroides	NA

-2.82	1.09	0.024	Bacteroidetes	Bacteroidia	Bacteroidales	Rikenellaceae	Alistipes	NA
-3.29	0.87	0.001	Bacteroidetes	Bacteroidia	Bacteroidales	Bacteroidaceae	Bacteroides	NA
-3.38	1.45	0.043	Firmicutes	Negativicutes	Selenomonadales	Veillonellaceae	Veillonella	NA
-1.65	0.55	0.008	Firmicutes	Negativicutes	Selenomonadales	Veillonellaceae	Veillonella	rogosae
-6.61	1.16	< 0.001	Bacteroidetes	Bacteroidia	Bacteroidales	Bacteroidaceae	Bacteroides	NA
1.75	0.65	0.018	Firmicutes	Clostridia	Clostridiales	Peptostreptococcaceae	NA	NA
-2.88	0.87	0.003	Firmicutes	Clostridia	Clostridiales	Peptostreptococcaceae	NA	NA
2.14	0.88	0.034	Firmicutes	Clostridia	Clostridiales	Clostridiaceae	Clostridium	NA
-3.94	1.06	0.026	Firmicutes	Clostridia	Clostridiales	Lachnospiraceae	Blautia	NA
-1.57	0.55	0.012	Firmicutes	Clostridia	Clostridiales	Ruminococcaceae	Gemmiger	NA
-3.17	0.94	0.003	Firmicutes	Clostridia	Clostridiales	Clostridiaceae	Clostridium	NA
4.43	1.09	< 0.001	Firmicutes	Clostridia	Clostridiales	Lachnospiraceae	NA	NA
-2.23	0.58	0.001	Firmicutes	Clostridia	Clostridiales	Lachnospiraceae	Lachnoclostridium	indolis

Abbreviations: ASV, amplicon sequence variant; SE, standard error; FDR, false discovery rate; NA, not available

* Negative beta coefficients indicate higher ASV relative abundance in infants not exposed to antibiotics in the second trimester, whereas positive beta coefficients indicate higher relative abundance in infants exposed to antibiotics in the second trimester.
